# A psychological intervention to promote acceptance and adherence to non-invasive ventilation in people with chronic obstructive pulmonary disease: study protocol of a randomised controlled trial

**DOI:** 10.1186/s13063-017-1802-1

**Published:** 2017-02-06

**Authors:** Eleonora Volpato, Paolo Banfi, Francesco Pagnini

**Affiliations:** 1 Department of Psychology, Università Cattolica del Sacro Cuore, Largo A. Gemelli, 1, 20123 Milan, Italy; 2 HD Respiratory Rehabilitation Unit, IRCCS Fondazione Don Carlo Gnocchi, Via Capecelatro 66, 20148 Milan, Italy; 3 Department of Psychology, Harvard University, Cambridge, MA USA

**Keywords:** Chronic obstructive pulmonary disease (COPD), Non-invasive ventilation (NIV), Acceptance, Adherence, Clinical psychology, Health psychology, Health economics

## Abstract

**Background:**

People with chronic obstructive pulmonary disease (COPD) sometimes experience anxiety, depression and comorbid cognitive deficits. Rather than being merely a consequence of symptom-related physical impairments these additional problems may be part of the clinical course of the condition. The relationship between the physical and psychological aspects of the condition is illustrated by the patterns of use of non-invasive ventilation (NIV); NIV is often rejected or used inappropriately, resulting in clinical deterioration and an increase in health care costs.

The study aims to analyse the effects of psychological support on the acceptance of, and adherence to, NIV. The primary outcome will be a latent variable related to indices of use of NIV equipment and adherence to treatment regime; while survival rates and psychological variables will constitute the secondary outcomes.

**Methods:**

A two-arm randomised controlled trial will be conducted. We aim to recruit 150 COPD patients for whom NIV is indicated. The experimental group will receive a brief course of psychological support that will include counselling, relaxation and mindfulness-based exercises. In some cases, it will also include neuropsychological rehabilitation exercises. Support will be delivered via four to eight meetings at the HD Respiratory Rehabilitation Unit, at home or via telemedicine. Controls will receive standard care and watch educational videos related to the management of their disease.

**Discussion:**

This investigation will gain insight about the role of a psychological intervention as part of a treatment plan during the process of adaptation to NIV in COPD patients.

**Trial registration:**

ClinicalTrials.gov, ID: NCT02499653. Registered on 14 July 2015.

**Electronic supplementary material:**

The online version of this article (doi:10.1186/s13063-017-1802-1) contains supplementary material, which is available to authorized users.

## Background

Chronic obstructive pulmonary disease (COPD) is the fourth leading cause of death in the world [1], the seventh cause of disability-adjusted life years (DALYs) and the tenth cause of early death (measured as years of life lost (YLL)) [2]. This clinical condition involves an irreversible obstruction of the airways, associated with progressive and chronic inflammation of the lung tissue; it develops slowly and adversely affects both quality of life (QoL) and psychological wellbeing [3], leading to high levels of anxiety and depression [4, 5]. The latter two are also associated with fatigue, shortness of breath and an increase in symptom exacerbations [6], engendering a psychological vicious circle of negativity [7–9]. Systematic reviews state that the prevalence of depression ranges between 7% and 42% [10], whereas that of anxiety varieties between 10% and 19% [11].

Cognitive deficits, whether global or in specific domains, such as perception, memory, attention [12–14], learning, visuospatial and construction abilities and language skills [15, 16], can also have an impact on functional, social, emotional and communication skills [17]. Moreover, COPD is associated with a significant risk of developing mild cognitive impairment, especially in patients older than 70 years [18].

Preliminary evidence suggests that it is necessary to pay attention to psychological factors in both therapy and rehabilitation [19–21]. Psychological changes may be a consequence of physical symptoms, but they may also influence the course of the disease, as in other chronic conditions [22].

Patterns of acceptance of non-invasive ventilation (NIV) are examples of the interaction of psychological and physical factors. Despite the fact that NIV can produce significant clinical improvement in their condition, patients often reject it or fail to use it appropriately. NIV rejection or its inappropriate use results in worse clinical outcomes and increased health care costs. Appropriate use of NIV is crucial, given that adherence to medication decreases over time and is inversely related to the number of drugs prescribed [23, 24]. Acceptance and adherence to NIV treatment can reduce hospital admissions and physician visits [25]. Using NIV requires the patient to make some behavioural and lifestyle changes, such as changes in daily routine to allocate time for ventilation, and this may reduce adherence to the treatment [24]. Treatment adherence may also be adversely affected by the use of different drugs and devices, or because patients receive inadequate education about the illness and associated morbidities from their physician [26, 27].

Clinical experience suggests that patients with cognitive deficits [12, 28, 29] or psychological problems, such as anxiety and depression, are less likely to accept and comply with NIV regimes; however, there has been little research on this. The few available studies focussed on early NIV failure (defined as the need for endotracheal intubation or death within 24–48 h of starting NIV) [30, 31], quantifying adherence [32] and largely neglected predictors of nonadherence [33]. No study has identified the individual psychological factors that increase the risk of rejection or improper use of NIV. Previous studies suggest that COPD patients who clearly demonstrated a lower survival benefit from the NIV usage exhibit worse average daily NIV adherence, because of the imbalance between the constraints that NIV imposes and the improvement that patients’ perceive in their condition [34]. Ventilator settings are not easy to adjust and they influence sleep quality, which has spill-over effects on daytime life [34, 35]. The effects of psychological support in promoting the machine’s acceptance have also not been adequately investigated.

### Objectives

The primary objective of the study is to evaluate the effects of psychological support on the acceptance of, and adherence to, NIV. Secondary aims include assessment of the change in QoL between baseline and after 3, 6 and 12 months following recruitment, identification of psychological and clinical factors that predict rejection or misuse of NIV, and factors that predict the success of the psychological intervention.

The primary outcome will be a latent variable related to indices of use of NIV equipment and adherence to treatment regime: the effects on change from baseline in acceptance to NIV in COPD patients at 3, 6 and 12 months as measured by weekly means and standard deviations of hours of use of NIV as recorded by the ventilator and changes in adherence defined as the difference between prescribed and effective NIV hours. Change in QoL will also be measured relative to baseline at 3, 6 and 12 months using the EuroQoL (EQ-5D) questionnaire, respiratory, functional and biomedical parameters. Survival rates after starting the adaptation to NIV and psychological variables will be considered as secondary outcomes.

As well as evaluating clinical and psychological variables we will also evaluate econometric outcomes. The study will explore the potential impact of the psychological intervention on the cost to the Italian welfare system of providing health care for patients with COPD. These assessments will be based on standard econometric indices (e.g. quality-adjusted life years (QALYs); DALYs).

Finally, semistructured interviews will be conducted to gain insight into patients’ perceptions of NIV.

## Methods

### Informed consent and ethical considerations

The study was approved by the Ethics Committee of the IRCCS Fondazione Don Carlo Gnocchi. The Study was registered at ClinicalTrials.gov, ID: NCT02499653. Amendments will be submitted to both the Ethics Committee and the Clinical Trials Register. All subjects will provide written, informed consent before the study begins. In particular, a psychologist will discuss the trial with patients in light of the information provided in the Consent Form and in Information Sheets. Patients will then be able to have an informed discussion with the participating consultant. The psychologist will obtain written consent from patients who are willing to participate in the trial.

All study-related information will be stored securely at the study site. All participant information will be stored in locked file cabinets in areas with limited access at Fondazione Don Carlo Gnocchi, Milan. All laboratory specimens, reports, data collection, process and administrative forms will be identified by a coded ID (identification) number only to maintain participant confidentiality. All records that contain names or other personal identifiers, such as Locator Forms and Informed Consent Forms, will be stored separately from study records identified by code number. All local databases will be secured with password-protected access systems. Participants’ study information will not be released outside of the study without the written permission of the participants. Moreover, the principal investigators will be the only persons allowed direct access to the data sets, and will also have access to other sites’ data by request. To ensure confidentiality, data dispersed to project team members will be blinded of any identifying participant information.

### Subjects and setting

The study will be conducted in a Clinical and Research Centre, HD Respiratory Rehabilitation Unit, Fondazione Don Carlo Gnocchi, IRCCS Santa Maria Nascente, Milan (Italy), a centre chosen based on documentation of, among other things, patient availability.

We aim to recruit 150 patients with COPD who have been recommended to use NIV based on the GOLD criteria (from moderate to severe) [36, 37].

The *inclusion criteria* are as follows: age 18 years or older; inpatients or outpatients; with moderate (GOLD 2–50% ≤ FEV_1_ < 80% predicted) to severe (GOLD 3–30% ≤ FEV_1_ < 50% predicted) COPD.

The *exclusion criteria* are as follows: refusal of consent; pregnancy; oncological history or history of psychiatric/mental disorders, according to *Diagnostic and Statistical Manual of Mental Disorders, fifth edition* (DSM-V) criteria [38, 39], with the exception of anxiety and depression, as indicated in the patient’s clinical record; immunosuppressive disease as the principal condition; stable use of antidepressant drugs.

### Design and interventions

**Fig. 1:**
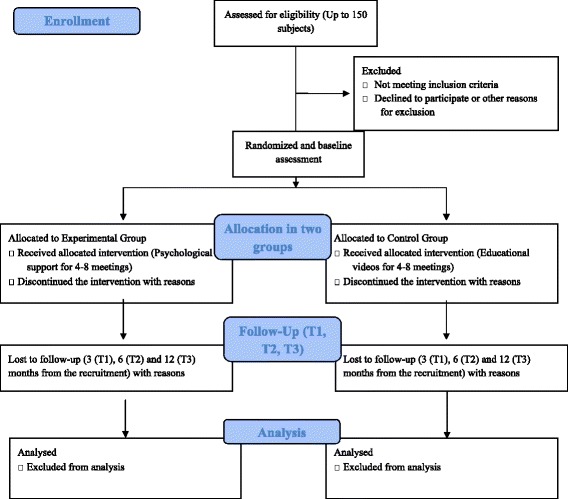
Flowchart of the study design

This study is a randomised control trial (RCT) (for the flowchart of the study design, see Fig. 1).

Subjects in the *experimental group* will receive a brief psychological intervention in addition to standard. This will include counselling and psychological support, mindfulness-based cognitive restructuring exercises [40] and relaxation training [41]. The intervention will also include neuropsychological rehabilitation exercises if required; this will be decided on a case-by-case basis, using clinical notes and the Addenbrooke’s Cognitive Examination (ACE-R) and Confusion Assessment Method (CAM) scores. Each patient’s psychological needs will be assessed at the beginning of the psychological intervention, paying special attention to aspects related to the disease (e.g. perception, understanding, awareness of symptoms). Thereafter, the psychological intervention will give more importance to the risks, doubts and fears arising from use of NIV. The intervention will consist of cognitive reframing exercises, relaxation training and, if necessary, neuropsychological rehabilitation exercises. Specifically, these intervention components extend from lowering arousal to providing disease information and management in order to understand better how to cope with NIV and with COPD symptoms, to an emotionally supportive environment in which subjects can address their fears and anxieties, as well as behavioural and cognitive coping strategies. The duration of the intervention will vary from a minimum of four weekly sessions to a maximum of eight according to the needs of the patient. Each session will last about 30–45 min. Sessions will be conducted in a hospital setting, or at the patients’ house or via telemedicine if patients are unable to attend the hospital.

Subjects assigned to the *control group* will spend six sessions watching videos related to the management of their disease (sessions will last about 30 min).

**Fig. 2:**
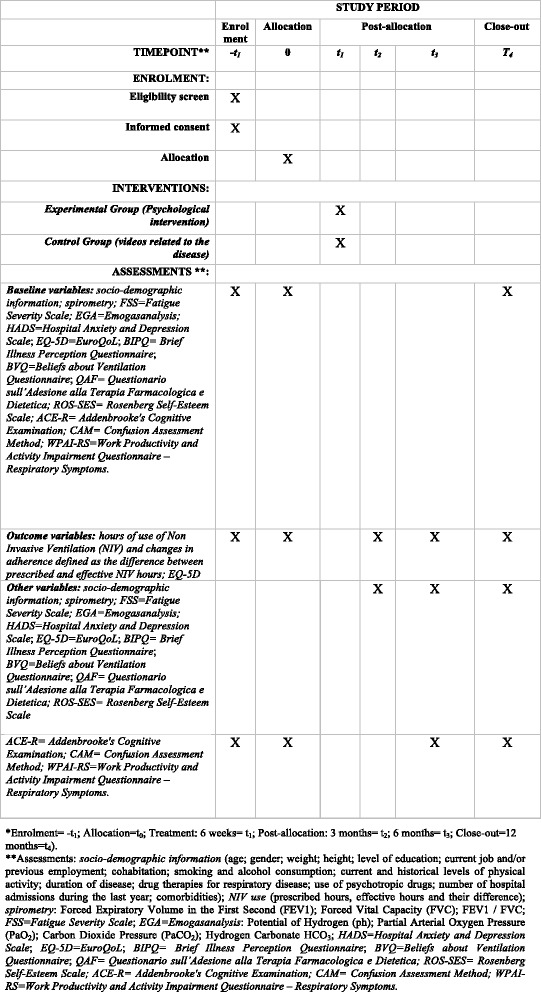
Study procedure: schedule of enrolment, interventions and assessments, reflecting the Standard Protocol Items: Recommendations for Interventional Trials (SPIRIT) figure

The psychologists who conduct the intervention will be blind to the assessments and vice versa (for the study procedure: schedule of enrolment, interventions and assessments, which reflects the Standard Protocol Items: Recommendations for Interventional Trials (SPIRIT) figure, see Fig. 2).

### Sample size

Based on statistical analysis, preventive inherent power and sample size [40] we anticipate that the study will require 150 subjects. We will recruit between 128 subjects (statistical power of 0.80; *α* = 0.05; 1 − *β* = 0.80; *t* (126) =1.97) and 172 subjects (statistical power equal to 0.90; *α* = 0.05; 1 − *β* = 0.90; *t* (170) = 1.97). The inherent power also emphasises that such analyses have been carried out using data for a scientific study that have a comparable primary outcome [33].

### Randomisation

#### Sequence generation

The patients will be randomised through a simple method, extracting a unit from the population using a method which guarantees the causality of the extractions (https://www.random.org).

#### Allocation concealment and implementation

Randomisation will be implemented using a computer system and the sequence will be concealed using a centralised service, since this cannot be subverted by either investigators or assessors and provides independent verification preventing the investigators to know the allocation sequence in advance until subjects are assigned to groups. We will calculate the dropout rate for both groups.

### Blinding (masking)

Subjects will not know in advance to which group they will be assigned and that the kind of treatment between control and experimental groups is different. Assessors will be blind to group allocation. A debriefing will be provided at the end of the study: the psychologist who carried out the intervention will debrief with the patients and will send them a report about the study’s findings.

### Assessment

#### Sociodemographic variables

At the first assessment we will collect sociodemographic information (age, gender, weight, height, level of education, current job and/or previous employment, cohabitation, smoking and alcohol consumption, current and historical levels of physical activity, duration of disease, drug therapies for respiratory disease, use of psychotropic drugs, number of hospital admissions during the last year, exacerbations and comorbidities).

#### Medical and clinical assessment

This will cover:

- NIV use (prescribed hours, effective hours and their difference)
- The effects on change from baseline in acceptance of NIV in COPD patients as measured by weekly means and standard deviations of hours of use of NIV as recorded by the ventilator
- Changes in adherence defined as the difference between prescribed and effective NIV hours
- Survival rate at different time after starting the adaptation to NIV, reported in terms of months
- Simple spirometry: forced expiratory volume in the first second (FEV1); forced vital capacity (FVC); FEV1/FVC
- Fatigue, measured by the Fatigue Severity Scale (FSS) [42]
- Emogasanalysis (EGA): potential of hydrogen (pH); partial pressure of oxygen in arterial blood (PaO2); partial pressure of carbon dioxide in arterial blood (PaCO2); hydrogen carbonate concentration (HCO3)

#### Psychological assessment

##### Anxiety and depression

Anxiety and depression will be assessed using the 14-item Hospital Anxiety and Depression Scale (HADS) [43]. Responses refer to the previous week and are given using a four-point Likert scale. The scale is designed to assess the severity of anxiety and depression simultaneously; it is organised into two 7-item subscales, HADS-A and HADS-D, which generate separate subscores for anxiety and depression, respectively.

##### Quality of life

QoL will be assessed using the EuroQoL (EQ-5D) [44, 45], a short, simple questionnaire consisting of two sections. The first comprises five health-related items (covering mobility, self-care, usual activities, pain/discomfort, anxiety/depression), and is used to assess the severity of health problems. The second section comprises a Visual Analogue Scale – a representation of a thermometer with graduated markings which respondents use to indicate their perception of their current health status (0 = ‘worst possible health state’ to 100 = ‘best possible health state’).

##### Representation of the disease

The representation that the patient has of their disease will be assessed using the Brief Illness Perception Questionnaire (BIPQ), developed by Broadbent and colleagues [46, 47]. This can be viewed as a practical application of the theory of self-regulation. Items can be organised into three categories: (1) identity of the disease (14 items), (2) opinion of the disease (38 items) and (3) causes of the disease (18 items). Responses are given using a five-point Likert scale (1 = ‘strongly disagree’ to 5 = ‘strongly agree’).

##### Representation of proposed therapies

This assessment will be based on a modified version of the Beliefs about Medicines Questionnaire (BMQ) developed by Horne and colleagues [48]. This tool is used to assess patients’ beliefs about medication. It consists of two scales: Specific Beliefs and General Beliefs. We will use a modified version of the BMQ designed to capture beliefs about NIV.

##### Therapeutic compliance

This will be assessed using the Questionario sull’Adesione alla Terapia Farmacologica e Dietetica (QAF) [49]. The QAF consists of 19 items to which responses are given using a four-point Likert scale (1 = ‘this is always true of me’; 2 = ‘this is often true of me’; 3 = ‘this is almost never true of me’; 4 = ‘this is never true of me’). Some of the items in this questionnaire are modifications of items of the Medication Adherence Rating Scale (MARS) which is used in psychiatry to assess adherence to drug therapy. The items were compiled and assorted randomly through extraction.

##### Self-esteem

Self-esteem will be assessed with the 10-item Rosenberg Self-Esteem Scale (RSES) [50]; responses are given on a four-point Likert scale (1 = ‘strongly agree’ to 4 = ‘strongly disagree’). The scale provides an indication of overall self-esteem and sense of self-worth.

#### Cognitive assessment

##### Addenbrooke’s Cognitive Examination (ACE-R)

The ACE-R [51] is a brief cognitive test that assesses five aspects of cognition: attention/orientation (AO), memory (M), verbal fluency (F), language (L) and visuospatial abilities (VS). Administration of the ACE-R takes 15 min on average.

##### Confusion Assessment Method (CAM)

This tool will enable us to evaluate features considered to be of great diagnostic importance, namely: acute onset, attentional deficits, disorganisation of thought, altered level of consciousness, disorientation, memory deficits, disorders of perception, psychomotor agitation, psychomotor retardation and disturbance of the sleep-wake cycle [52].

#### Econometric assessment

We will analyse the direct (e.g. supply of equipment, maintenance tasks, etc.) and indirect (e.g. cost of hospitalisation for relapse, additional care, indirect social costs, etc.) costs of the management of COPD in our sample, referring to clinical notes and medical files. Changes in QoL and life expectancy data will be used to estimate QALYs [53, 54] and DALYs [55] and, by comparing our data with international norms, we will gain insight into the sustainability and cost-efficiency of the psychological intervention. We also plan to use econometric tools such as the Work Productivity and Activity Impairment Questionnaire – Respiratory Symptoms (WPAI-RS) [56, 57].

The medical assessment will take about 30 min and the psychological assessment about 45–50 min. The order of administration of the questionnaires and tests will be randomised on a per subject basis.

Finally, semistructured interviews (qualitative data) will be carried out to provide insight into subjects’ views on use of the NIV equipment.

### Recruitment and duration of the study

The study is expected to last 3 years. After the pulmonary clinical visit all patients who meet the inclusion criteria will be asked to join the study. If they agree to do so they will be asked to sign a form to indicate their informed consent to participate. After this they will undergo psychological assessment (baseline, T0). Follow-up assessments will be carried out 3, 6 and 12 months after recruitment (T1, T2 and T3, respectively) using the same procedure.

### Proposed analyses

Descriptive analyses will be performed for all study variables. We will assess the effect of the interaction between treatment (experimental versus control) and time (three repeated measures) on primary outcome variables (acceptance of treatment; adherence to treatment). Missing data will be imputed with multiple imputation techniques [58, 59].

Before-after differences will be analysed with appropriate within-group tests (paired samples *t* test; Wilcoxon test). Multiple regression will be used to assess the influence of baseline variables on outcome indicators and identify predictor variables.

The analysis will be performed using the intention-to-treat procedure. The significance level will be 5% (*p* ≤ 0.05). Data analysis will be performed using SPSS software.

Data collection and analysis will begin in month 1 and will last for 24 months, i.e. until 1 month after the last follow-up data have been collected.

We will report reasons for withdrawal for each randomisation group and compare the reasons qualitatively. The effect that any missing data might have on results will be assessed via sensitivity analysis of augmented data sets. Dropouts (essentially, participants who withdraw consent for continued follow-up) will be included in the analysis by modern imputation methods for missing data.

For more details about the trial, refer to the SPIRIT Checklist (Additional file 1).

## Discussion

We anticipate some limitations or threats to the efficacy of the study protocol. First, the rigorous RCT design will be in contrast with the ‘single-patient’ approach. As part of the ‘single-patient’ approach patients will be free to choose the setting in which they receive the intervention; this may make it difficult to achieve an even distribution of settings. Third, patients who choose to use telemedicine might face difficulty accessing the intervention owing to technical problems.

Despite these limitations the design has many strengths. It investigates a form of psychological support specifically designed to facilitate adaptation to NIV. Moreover, the intervention will be tailored to patients’ needs and clinical status. The study protocol is projected in order to reduce barriers to participation such as disabilities (i.e. movements, dyspnoea) and practical barriers (daily activities and habits). Finally, to reduce the Hawthorne effect the experimental condition will be compared with an active control group, which will watch films related to the management of many aspects of COPD.

This study has the potential to demonstrate the viability of an easy-access intervention based on a ‘single-patient’ approach that can be used in several settings to facilitate adaptation to NIV and improve the QoL of COPD patients. This study protocol describes the development of the study design and the related intervention. If the hypothesis is confirmed, the intervention will have positive effects on physical and psychological wellbeing, and consequently reduce the costs of health care for COPD patients.

### Trial status

Recruitment is ongoing.

## Additional file

Additional file 1: SPIRIT 2013 Checklist: recommended items to address in a clinical trial protocol and related documents. (DOC 122 kb)

## Abbreviations

ACE-R: Addenbrooke’s Cognitive Examination
BIPQ: The Brief Illness Perception Questionnaire
CAM: Confusion Assessment Method
COPD: Chronic obstructive pulmonary disease
DALY: Disability-adjusted life year
EQ-5D: Hospital Anxiety and Depression Scale
FEV_1_: Forced expiratory volume in the first second
FSS: Fatigue Severity Scale
FVC: Forced vital capacity
GOLD: Global Initiative for Chronic Obstructive Pulmonary Disease
HADS: Hospital Anxiety and Depression Scale
MARS: Medication Adherence Rating Scale
NIV: Non-invasive ventilation
PaCO_2_: Partial pressure of carbon dioxide in arterial blood
PaO_2_: Partial pressure of oxygen in arterial blood
pH: Potential of hydrogen indicator
QAF: Questionnaire of pharmacological adherence to therapy and dietetics
QALY: Quality- adjusted life years
RCT: Randomised control trial
RSES: Rosenberg Self-Esteem Scale
WPAI-RS: Work Productivity and Activity Impairment Questionnaire – Respiratory Symptoms
YLL: Year of life lost

## References

[1] World Health Organization. The world health report 2000: health systems: improving performance. World Health Organization. 2000.

[2] Institute for Health Metrics and Evaluation. The global burden of disease: generating evidence, guiding policy. 2013.

[3] Fabbri LM, Hurd SS (2003). Global strategy for the diagnosis, management and prevention of COPD: 2003 update. Eur Respir J.

[4] Wells KB (1989). Detection of depressive disorder for patients receiving prepaid or fee-for-service care. Results from the Medical Outcomes Study. JAMA.

[5] Williams JW, Mulrow CD, Kroenke K, Dhanda R, Badgett RG, Omori D, Lee S. Case-finding for depression in primary care: a randomized trial. Am J Med. 1999;106(1):36–43. 10.1016/s0002-9343(98)00371-4 10320115

[6] Doyle T, Palmer S, Johnson J, Babyak MA, Smith P, Mabe S (2013). Association of anxiety and depression with pulmonary-specific symptoms in chronic obstructive pulmonary disease. Int J Psychiatry Med.

[7] Di Marco F, Verga M, Reggente M, Maria Casanova F, Santus P, Blasi F (2006). Anxiety and depression in COPD patients: the roles of gender and disease severity. Respir Med.

[8] Gift AG, Plaut SM, Jacox A. Psychologic and physiologic factors related to dyspnea in subjects with chronic obstructive pulmonary disease. Heart Lung: J Crit Care. 1986;15(6):595–601. 3639857

[9] Gudmundsson G, Gislason T, Janson C, Lindberg E, Suppli Ulrik C, Brøndum E (2006). Depression, anxiety and health status after hospitalisation for COPD: a multicentre study in the Nordic countries. Respir Med.

[10] van Ede L, Yzermans CJ, Brouwer HJ (1999). Prevalence of depression in patients with chronic obstructive pulmonary disease: a systematic review. Thorax.

[11] Maurer J (2008). Anxiety and depression in COPD: current understanding, unanswered questions, and research needs. Chest J.

[12] Dodd JW, Getov SV, Jones PW (2010). Cognitive function in COPD. Eur Respir J.

[13] Incalzi RA, Gemma A, Marra C, Muzzolon R, Capparella O, Carbonin P (1993). Chronic obstructive pulmonary disease. an original model of cognitive decline. Am Rev Respir Dis.

[14] Schou L, Østergaard B, Rasmussen LS, Rydahl-Hansen S, Phanareth K (2012). Cognitive dysfunction in patients with chronic obstructive pulmonary disease—A systematic review. Respir Med.

[15] Antonelli-Incalzi R, Corsonello A, Trojano L, Pedone C, Acanfora D, Spada A (2007). Screening of cognitive impairment in chronic obstructive pulmonary disease. Dement Geriatr Cogn Disord.

[16] Etnier JL, Berry M (2001). Fluid intelligence in an older COPD sample after short- or long-term exercise. Med Sci Sports Exerc.

[17] Andreou G, Vlachos F, Makanikas K (2014). Effects of chronic obstructive pulmonary disease and obstructive sleep apnea on cognitive functions: evidence for a common nature. Sleep Disord.

[18] Singh B, Mielke MM, Parsaik AK, Cha RH, Roberts RO, Scanlon PD (2014). A prospective study of chronic obstructive pulmonary disease and the risk for mild cognitive impairment. JAMA Neurol.

[19] Phillips D, Pagnini F. A mindful approach to chronic illness. In: In A. Le, C. T. Ngnoumen, E. Langer, editors. The Wiley Blackwell handbook of mindfulness. London: Wiley-Blackwell. 2014. p. 852–63.

[20] Lacasse Y, Goldstein R, Lasserson TJ, Martin S. Pulmonary rehabilitation for chronic obstructive pulmonary disease. Cochrane Database Syst Rev. 2006;4(4). 10.1002/14651858.CD003793.pub2 17054186

[21] Celli BR (1995). Pulmonary rehabilitation in patients with COPD. Am J Respir Crit Care Med.

[22] Pagnini F, Bosma CM, Phillips D, Langer E. Symptom changes in multiple sclerosis following psychological interventions: a systematic review. BMC Neurol. 2014;14(1):222. 10.1186/s12883-014-0222-z PMC4253984 25433519

[23] Claxton AJ, Cramer J, Pierce C (2001). A systematic review of the associations between dose regimens and medication compliance. Clin Ther.

[24] Restrepo RD, Alvarez MT, Wittnebel LD, Sorenson H, Wettstein R, Vines DL (2008). Medication adherence issues in patients treated for COPD. Int J Chron Obstruct Pulmon Dis.

[25] Balkrishnan R, Christensen DB (2000). Inhaled corticosteroid use and associated outcomes in elderly patients with moderate to severe chronic pulmonary disease. Clin Ther.

[26] Chryssidis E, Frewin DB, Frith PA, Dawes ER. Compliance with aerosol therapy in COPD. N Z Med J. 1981;250:375–7. 6948205

[27] Dolce JJ, Crisp C, Manzella B, Richards JM, Hardin JM, Bailey WC (1991). Medication adherence patterns in chronic obstructive pulmonary disease. Chest.

[28] Antonelli Incalzi R, Marra C, Giordano A, Calcagni ML, Cappa A, Basso S (2003). Cognitive impairment in chronic obstructive pulmonary disease—a neuropsychological and SPECT study. J Neurol.

[29] Lareau SC, Yawn B (2010). Improving adherence with inhaler therapy in COPD. Int J Chron Obstruct Pulmon Dis.

[30] Ko BS, Ahn S, Lim KS, Kim WY, Lee Y-S, Lee JH (2015). Early failure of noninvasive ventilation in chronic obstructive pulmonary disease with acute hypercapnic respiratory failure. Intern Emerg Med.

[31] Ozyilmaz E, Ugurlu AO, Nava S. Timing of noninvasive ventilation failure: causes, risk factors, and potential remedies. BMC Pulm Med [Internet]. 2014;14. Available from: http://bmcpulmmed.biomedcentral.com/articles/10.1186/1471-2466-14-19. Accessed 16 Dec 2016. 10.1186/1471-2466-14-19 PMC3925956 24520952

[32] Hess DR. The growing role of noninvasive ventilation in patients requiring prolonged mechanical ventilation. Respir Care. 2012;57(6):900–20. 10.4187/respcare.01692 22663966

[33] Leiva-Fernández J, Leiva-Fernández F, García-Ruiz A, Prados-Torres D, Barnestein-Fonseca P. Efficacy of a multifactorial intervention on therapeutic adherence in patients with chronic obstructive pulmonary disease (COPD): a randomized controlled trial. BMC Pulm Med. 2014;14(1):70. 10.1186/1471-2466-14-70 PMC4011779 24762026

[34] Borel J-C, Pepin J-L, Pison C, Vesin A, Gonzalez-Bermejo J, Court-Fortune I (2014). Long-term adherence with non-invasive ventilation improves prognosis in obese COPD patients. Respirol Carlton Vic.

[35] Adler D, Perrig S, Takahashi H, Espa F, Rodenstein D, Pépin JL (2012). Polysomnography in stable COPD under non-invasive ventilation to reduce patient-ventilator asynchrony and morning breathlessness. Sleep Breath Schlaf Atm.

[36] Decramer M (2015). Global Strategies for the diagnosis, management, and prevention of chronic obstructive pulmonary disease: Global Initiative for Chronic Obstructive Lung Disease.

[37] GOLD. Global strategy for the diagnosis, management and prevention of COPD: Global Initiative for Chronic Obstructive Lung Disease (GOLD). 2016. Available from: http://www.goldcopd.org.

[38] Diagnostic APA. Statistical manual of mental disorders: DSM-5 (ed.). Washington, DC: American Psychiatric Association; 2013.

[39] Stein DJ, Phillips KA, Bolton D, Fulford KWM, Sadler JZ, Kendler KS. What is a mental/psychiatric disorder? From DSM-IV to DSM-V. Psychol Med. 2010;40(11):1759–65. 10.1017/S0033291709992261 PMC3101504 20624327

[40] Pagnini F, Philips D (2015). Being mindful about mindfulness. Lancet Psychiatry.

[41] Volpato E, Banfi P, Rogers SM, Pagnini F (2015). Relaxation techniques for people with chronic obstructive pulmonary disease: a systematic review and a meta-analysis. Evid-Based Complement Altern Med.

[42] Schwartz JE, Jandorf L, Krupp LB (1993). The measurement of fatigue: a new instrument. J Psychosom Res.

[43] Zigmond AS, Snaith RP (1983). The Hospital Anxiety and Depression Scale. Acta Psychiatr Scand.

[44] Szende A, Oppe M, Devlin NJ, EuroQol Group (2007). EQ-5D value sets: inventory, comparative review, and user guide.

[45] Balestroni G, Bertolotti G (2012). EuroQol-5D (EQ-5D): an instrument for measuring quality of life. Monaldi Arch Chest Dis.

[46] Broadbent E, Petrie KJ, Main J, Weinman J (2006). The brief illness perception questionnaire. J Psychosom Res.

[47] Pain D, Miglioretti M, Angelino E. Sviluppo della versione italiana del Brief-IPQ (Illness Perception Questionnaire, short version), strumento psicometrico per lo studio delle Rappresentazioni di Malattia. Psicol. Della Salute. 2006.

[48] Horne R, Weinman J (1999). Patients’ beliefs about prescribed medicines and their role in adherence to treatment in chronic physical illness. J Psychosom Res.

[49] Gerbino G, Dimonte V, Albasi C (2011). Adesione alla terapia del paziente in emodialisi. G Ital Nefrol.

[50] Robins RW, Hendin HM, Trzesniewski KH (2001). Measuring global self-esteem: Construct validation of a single-item measure and the Rosenberg Self-Esteem Scale. Personal Soc Psychol Bull.

[51] Mioshi E, Dawson K, Mitchell J, Arnold R, Hodges JR (2006). The Addenbrooke’s Cognitive Examination Revised (ACE-R): a brief cognitive test battery for dementia screening. Int J Geriatr Psychiatry.

[52] Inouye SK, van Dyck CH, Alessi CA, Balkin S, Siegal AP, Horwitz RI (1990). Clarifying confusion: the confusion assessment method. A new method for detection of delirium. Ann Intern Med.

[53] Murray CJL, Barber RM, Foreman KJ, Ozgoren AA, Abd-Allah F, Abera SF (2015). Global, regional, and national disability-adjusted life years (DALYs) for 306 diseases and injuries and healthy life expectancy (HALE) for 188 countries, 1990–2013: quantifying the epidemiological transition. Lancet.

[54] Williams A, Evans R, Drummond M (1987). Quality-adjusted life-years. Lancet.

[55] Murray CJ (1994). Quantifying the burden of disease: the technical basis for disability-adjusted life years. Bull World Health Organ.

[56] Ståhl E, Jansson SA, Jonsson AC, Svensson K, Lundbäck B, Andersson F. Health-related quality of life, utility, and productivity outcomes instruments: ease of completion by subjects with COPD. Health Qual Life Outcomes. 2003;1(1):18. 10.1186/1477-7525-1-18 PMC161803 12809558

[57] Reilly MC, Zbrozek AS, Dukes EM (1993). The validity and reproducibility of a work productivity and activity impairment instrument. Pharmacoeconomics.

[58] Groenwold RH, Donders ART, Roes KC (2012). Dealing with missing outcome data in randomized trials and observational studies. Am J Epidemiol.

[59] Groenwold RH, White IR, Donders ART (2012). Missing covariate data in clinical research: when and when not to use the missing-indicator method for analysis. Can Med Assoc J.

